# Qualitative Characterization of Neu5Gc-Containing N-Glycans in Commercial Cheddar Cheese Products Using Nano-UHPLC–ESI–MS/MS—A Pilot Study

**DOI:** 10.3390/ijms27104200

**Published:** 2026-05-08

**Authors:** Damir Mogut, Mona Goli, Cristian D. Gutierrez Reyes, Yehia Mechref, Anna Iwaniak

**Affiliations:** 1 Department of Food Biochemistry, Faculty of Food Science, University of Warmia and Mazury in Olsztyn, 10-719 Olsztyn, Poland; 2 Department of Chemistry and Biochemistry, Texas Tech University, Lubbock, TX 79409, USA

**Keywords:** cheese, food analysis, N-glycan, Neu5Gc, mass spectrometry, qualitative identification

## Abstract

N-Glycolylneuraminic acid (Neu5Gc) is a non-human sialic acid present in mammalian tissues and certain dairy products. Although humans cannot synthesize Neu5Gc, it can be metabolically incorporated from dietary sources, potentially leading to the generation of anti-Neu5Gc antibodies, and has been associated with chronic inflammatory responses. While Neu5Gc distribution has been extensively studied in red meat, its presence in processed dairy products remains insufficiently characterized. In this pilot study, nano ultra-high-performance liquid chromatography coupled with electrospray ionization–tandem mass spectrometry (nano-UHPLC–ESI–MS/MS) was used for qualitative characterization of Neu5Gc-containing N-glycans in two commercially available cheese products. Neu5Gc-positive N-glycans were identified in reduced-fat sharp cheddar cheese (CHE), whereas such structures were not observed in processed cheddar cheese slices (SLI). These findings suggest that dairy processing and formulation parameters may influence glycan composition and Neu5Gc identification in cheddar cheese. Further studies using quantitative approaches and a broader range of dairy matrices are warranted to elucidate how processing-related factors affect Neu5Gc availability and its potential implications for diet-associated inflammation.

## 1. Introduction

Complex food matrices of animal origin contain a wide range of structurally diverse glycans associated with proteins and lipids. Animal-derived foods, such as dairy products, represent particularly complex systems, as they undergo multiple biochemical and technological processes, including enzymatic activity, fermentation, heat treatment, and ripening, which can significantly influence glycan composition and structure [1]. Among these, glycans present on milk proteins or released as free oligosaccharides have gained increasing scientific attention due to their structural diversity and functional relevance [2].

One glycan-associated component of particular interest is N-glycolylneuraminic acid (Neu5Gc), a non-human sialic acid [3]. Although humans cannot synthesize Neu5Gc, dietary intake of red meat and dairy products serves as an exogenous source of this non-human sialic acid, which can be subsequently metabolically incorporated into cell surface and secreted glycans, glycoproteins, and glycolipids, while simultaneously eliciting polyclonal anti-Neu5Gc antibody responses [4]. Once incorporated, Neu5Gc may elicit circulating anti-Neu5Gc antibodies, which have been associated with inflammatory processes, atherosclerosis, and cancer biology [5]. According to Padler-Karavani et al. [6], the role of anti-Neu5Gc antibodies in cancer is contradictory—they can either stimulate or suppress tumor progression depending on their dose. Moreover, some of these antibodies may act as carcinoma biomarkers. Overall, the precise function of anti-Neu5Gc antibodies remains an area of active debate [6].

An important but underexplored aspect of Neu5Gc biology concerns the fate of glycans during digestion. While many carbohydrate structures are enzymatically degraded in the gastrointestinal tract, emerging evidence suggests that some glycans—or their metabolic derivatives—may escape complete digestion and enter systemic circulation, where they can be incorporated into human glycoconjugates [7]. This raises the possibility that dietary Neu5Gc may derive not only from intact foodborne glycans but also from intermediary molecules produced during digestion. Understanding these mechanisms is essential for clarifying the relationship between dietary Neu5Gc exposure and its immunological consequences.

A review of the available literature indicates that research on Neu5Gc has predominantly focused on its immunological properties, chemical characteristics, and dietary exposure, particularly in the context of red meat and animal models. The presence of Neu5Gc in dairy products, including cheese, has been reported in broader dietary and food composition studies; for example, Neu5Gc has been detected in Cheddar cheese in supplementary datasets of studies primarily addressing inflammation and cancer biology, as well as in food surveys based on bulk sialic acid quantification methods [8]. More recently, Rinaldi et al. reported the presence of Neu5Gc in various commercial cheeses using quantitative analysis of total sialic acid content [9]. However, these studies relied on bulk measurements and did not include qualitative structural characterization of Neu5Gc-containing N-glycans, nor did they examine Neu5Gc at the level of individual N-glycan compositions in cheddar cheese matrices. Consequently, detailed qualitative characterization of Neu5Gc-containing N-glycans in cheese remains underexplored.

Qualitative analytical techniques are becoming essential tools in food science and industry for verifying authenticity, detecting fraud, and ensuring traceability throughout the food supply chain [10]. A brief description of qualitative analysis provided by the International Union of Pure and Applied Chemistry (IUPAC) characterizes it as the identification or classification of substances (including foods) based on chemical and physical properties. This concept applies not only to single compound identification but also to broader food chemistry applications, including authenticity testing, fraud detection, and process control [10].

Chromatography, described by Debnath et al. [11] as “a widely utilized method for separating, purifying, and identifying various substances,” has been broadly applied for the identification of glycans in biological samples [12]. For example, Guerrero-Flores et al. [12] employed LC–MS/MS for the simultaneous quantification of N-acetyl-D-mannosamine (ManNAc) and N-acetylneuraminic acid (Neu5Ac) in 155 samples of human blood sera. She et al. [13] utilized tandem mass spectrometry coupled with chromatographic separation for highly sensitive characterization of non-human glycan structures, including Neu5Gc, in monoclonal antibody drugs. Furthermore, Zarei et al. [14] demonstrated the applicability of online nano-HPLC coupled with quadrupole time-of-flight mass spectrometry (QToF MS) and tandem MS for the separation and identification of Neu5Ac- and Neu5Gc-containing gangliosides, highlighting the potential of this approach for glycolipidomics screening of animal cell lines.

Thus, the aim of this study was to investigate the qualitative characteristics of Neu5Gc-containing N-glycans in two commercially available cheese products: sharp cheddar cheese and pasteurized processed cheddar cheese slices, using ultra-high-resolution nano-UHPLC–ESI–MS/MS, in order to demonstrate the applicability of this analytical approach to complex dairy matrices.

## 2. Results and Discussion

Commercially available cheese products, i.e., sharp cheddar cheese and pasteurized processed cheese slices, were obtained from Lucerne Dairy Farms and purchased in a local Walmart store in Lubbock, TX, USA. Sharp cheddar was selected as a representative aged dairy product with a relatively simple ingredient composition and well-characterized fermentation profile, making it suitable for proof-of-concept glycomic analysis [10,15].

Citing the words by Lehotay et al. [16], “mass spectrometry (MS) is generally assumed to be the gold standard for qualitative methods, and its results are typically unquestioned”. The cheese samples were analyzed using a nano-UHPLC–ESI–MS/MS system (Orbitrap Fusion Lumos, Thermo Fisher Scientific, San Jose, CA, USA), and data acquisition and processing were performed in Xcalibur (Thermo Fisher Scientific). N-glycan assignments were made using MultiGlycan software (MultiGlycan_ESI Quantification 1.4.0) [17].

**Figure 1:**
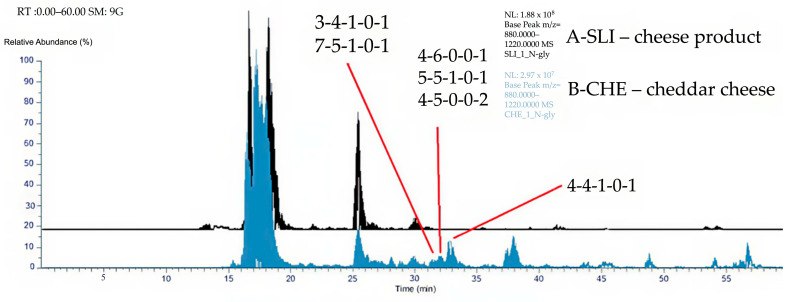
Total ion chromatograms of analyzed samples. Abbreviations: A: chromatogram of processed cheddar cheese slices (black; SLI), B: chromatogram of cheddar cheese (blue; CHE).

Figure 1 shows the MS analysis of the cheese samples within a retention time window of 0–60 min and an *m*/*z* range of 880–1220. Total ion chromatograms (TICs) were smoothed using a nine-point Gaussian algorithm. Neu5Gc-containing N-glycans eluted primarily between 31.0 and 36.2 min, with the most abundant species identified at 33.30 ± 0.03 min. The identities of all observed Neu5Gc-positive structures are summarized in Table 1, including average retention times (RT, min), peak abundances, observed *m*/*z* values, proposed glycan compositions, adduct types, and calculated monoisotopic masses. Data represent mean ± SD from triplicate injections where applicable.

Peak area variability among Neu5Gc-containing N-glycans in the cheddar cheese (CHE) sample ranged from ~30–40% SD. This magnitude of variability is typical for glycomic studies and likely reflects minor differences in sample handling and injection. The ±5 ppm mass accuracy specified in the methods ensured precise glycan identification. Although the quantitative variation introduces some uncertainty, the qualitative identification of Neu5Gc-containing species remains reliable. Nevertheless, the results should be interpreted with cautious optimism, as they represent an initial step toward further characterization of Neu5Gc-containing N-glycans not only in cheddar but also in a wider variety of cheeses. Such characterization should include, e.g., the use of internal standards for more robust quantitative analysis of these molecules. Future work incorporating internal standards would enable better normalization and improve quantitative reproducibility.

**Figure 2:**
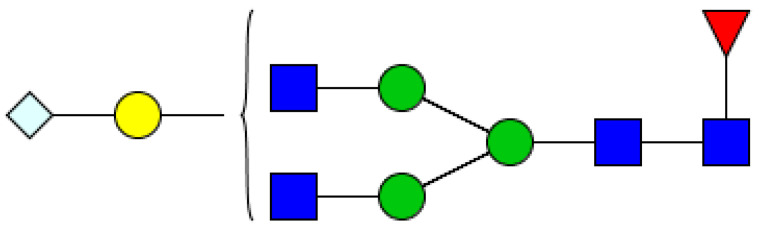
Proposed structure of the most abundant Neu5Gc-containing N-Glycan identified in cheddar cheese (CHE): HexNAc_4_Hex_4_Fuc_1_Neu5Gc_1_ (*m/z* 1213.1300 [M+2H]^2+^, RT = 33.30 min). Symbols (see Abbreviations) follow the Symbol Nomenclature for Glycans (SNFG): blue square, GlcNAc; yellow circle, galactose; green circle, mannose; red triangle, fucose; light blue diamond, Neu5Gc. (https://glycosmos.org/glycans/graphic/SugarDrawer (accessed on 30 January 2026); [18]).

High-resolution MS analysis confirmed the presence of multiple Neu5Gc-containing N-glycans in the cheddar cheese extract. The most abundant glycan—eluting at 33.30 ± 0.03 min with an *m*/*z* of 1213.1300 ([M+2H]^2+^) and a peak area of 3,317,978 ± 1,277,497—was consistent with a core-fucosylated, biantennary complex-type N-glycan (HexNAc_4_ Hex_4_ Fuc_1_ Neu5Gc_1_). Its high signal intensity indicates that Neu5Gc is not a minor contaminant but a notable structural component of the cheddar N-glycome (Figure 2). In contrast, Neu5Gc-containing N-glycans were absent from the processed cheddar cheese slice (SLI) sample.

It is well-known that milk and dairy products contain Neu5Gc [9]. To date, it has been demonstrated that milk origin, chemical composition, and cheesemaking technology affect the content of glycans [9]. To the best of our knowledge, processed cheeses have not yet been investigated as potential sources of Neu5Gc-containing N-glycans. Briefly, the production of processed cheese involves blending shredded natural cheeses of different types and degrees of maturity with emulsifying salts. The blend is heated under partial vacuum with constant agitation until a homogeneous mass is obtained. The mixture may also contain additional dairy and non-dairy ingredients [19]. The absence of Neu5Gc-containing N-glycans in the analyzed processed cheese slices was observed under the applied analytical conditions. However, this finding may reflect multiple factors, including differences in product formulation, relative proportions of milk-derived ingredients, or processing practices. Further studies using a broader range of samples and detailed compositional analyses are required to elucidate the mechanisms underlying Neu5Gc reduction or absence in dairy products. To gain a comprehensive understanding of the occurrence and behavior of Neu5Gc-containing N-glycans in food matrices, future research should encompass a broader range of food products derived from various sources and manufacturers. This work aimed at the qualitative characterization of Neu5Gc in cheddar cheese. Our qualitative analysis is the initial step for further characterization of Neu5Gc in food products, such as quantification. However, before quantification, one should ask a question: “What is the identified substance?” After its identification, the next step is to determine the quantity of the substance in the sample. According to Schmitz and Meckelmann [20], identification (put as first) and quantification (put as second) are the core of modern analytical chemistry.

To recapitulate, this study provides qualitative identification of Neu5Gc-containing N-glycans in cheddar cheese, while potential dietary and immunological implications remain tentative and require dedicated quantitative and biological investigation. Existing literature suggests, primarily in the context of red meat consumption, that Neu5Gc, as a non-human sialic acid, can be incorporated into human glycoproteins following ingestion, where it may be recognized by circulating anti-Neu5Gc antibodies. While Neu5Gc does not typically elicit acute IgE-mediated allergic responses, chronic exposure has been associated with low-grade inflammation (“xenosialitis”) and has been discussed in the context of metabolic and degenerative diseases such as atherosclerosis, colorectal cancer, and type 2 diabetes [5,8,21]. Some studies have further proposed that anti-Neu5Gc immune responses may, under specific conditions, contribute to immune surveillance. However, this effect appears to be context-dependent and remains an area of ongoing debate [6].

Several limitations should be acknowledged. Only two cheese products were analyzed, limiting generalizability. Additionally, exoglycosidase digestion was not performed, preventing confirmation of specific sialic acid linkage types (e.g., α2–3 vs. α2–6) [22]. Future studies should include enzymatic verification, standards spiking, and expanded sampling across cheese varieties, brands, and processing conditions.

Overall, these findings highlight the importance of qualitative characterization of food-derived glycans in complex dairy matrices. The identification of Neu5Gc-containing N-glycans in cheddar cheese, contrasted with their non-detection in the analyzed processed cheese slices, possibly suggests that product formulation and/or processing may modulate the presence of Neu5Gc-associated structures. While this observation does not allow definitive conclusions regarding processing mechanisms or dietary exposure, it supports the notion that technological and compositional factors may influence Neu5Gc occurrence in dairy products and warrants further investigation. Future research should focus on

- Quantifying Neu5Gc (if present in a sample) across a broader range of cheese varieties and manufacturers;
- Investigating the bioavailability, metabolic fate, and tissue incorporation of Neu5Gc after consumption;
- Evaluating its immunomodulatory effects in healthy and vulnerable populations.

Such complex work will be pivotal for understanding the role of Neu5Gc in food safety, personalized nutrition, and immune-mediated health outcomes.

## 3. Materials and Methods

Cheddar cheese samples (brand: Lucerne Dairy Farms) were purchased from a local Walmart store in Lubbock, TX, USA, twice a month in October and November 2022. Table 2 summarizes the listed ingredients and nutritional information obtained directly from product packaging. The CHE sample corresponds to a naturally aged, reduced-fat sharp cheddar cheese produced through fermentation and ripening from pasteurized milk, starter cultures, salt, and enzymes. The SLI sample represents a pasteurized processed cheese product consisting of a cheese base combined with additional dairy-derived and functional ingredients and manufactured using high-temperature melting, emulsifiers, and melting salts. These differences in product type and formulation provide important context for the interpretation of the qualitative glycan profiles observed in this study.

### 3.1. Sample Preparation

All experiments were performed in triplicate. Cheese samples of each purchase were solubilized in 5% sodium deoxycholate (SDC) (Millipore Sigma, Burlington, MA, USA, Cat. No 264103) prepared in 50 mM ammonium bicarbonate (ABC) buffer (Merck KGaA, Darmstadt, Hessen, Germany, Cat. No A6141) at a final concentration of 1 mg/mL. Samples were incubated at 37 °C for 30 min, homogenized with 2.8 mm zirconium beads (OPS Diagnostics, Lebanon, NJ, USA) using a SPEX SamplePrep Geno-Grinder (Metuchen, NJ, USA) at 1750 rpm, and centrifuged at 4 °C, 1.5 krpm in an Allegra X-12R centrifuge (Beckman Coulter, Indianapolis, IN, USA). After removing the lipid layer, pellets were dried and resuspended in 5% SDC (Merck D6750) prepared in 10 mM phosphate-buffered saline (PBS; Merck 524650). The prepared samples were pooled, homogenized, and used for subsequent analyses.

Protein concentrations were measured using a Micro BCA Protein Assay Kit (Thermo Fisher Scientific, Waltham, MA, USA, Cat. No 23235). A total of 2.5 µg of protein was diluted in 50 µL ABC buffer. A 5 kDa molecular weight cut-off (MWCO) filtration was performed using an Amicon tube (Merck, Cat. No UFC903008). The >5 kDa retentate was denatured and digested with PNGase F (Merck, Cat. No G5166) using an enzyme-to-protein ratio of 1:5, followed by incubation at 37 °C for 18 h.

Following digestion, glycans were precipitated using 90% cold ethanol (Merck Cat. No 459844) and centrifuged at maximum speed for 2 min. The aqueous phase was collected and desalted by dialysis using a custom device fitted with a 500–1000 Da MWCO membrane (Spectra Por 131096, Vernon Hills, IL, USA).

Glycans were then reduced and permethylated following Zhou et al. [23]. Briefly, samples were reduced using 10 µL borane–ammonia complex (10 mg/mL) (Merck Cat. No A4505) at 60 °C for 1 h. Residual borate was removed by adding 500 µL methanol, followed by evaporation to dryness, repeated three times. Reduced glycans were resuspended in 30 µL DMSO, 1.2 µL water, and 20 µL iodomethane (Merck, Cat. No 67692), then loaded onto a sodium hydroxide bead-packed spin column (Harvard Apparatus, Holliston, MA, USA, Item No. 74-4420). The column was incubated at room temperature for 25 min, followed by the addition of another 20 µL iodomethane and a second incubation for 15 min. Samples were eluted by centrifugation at 1800 rpm for 2 min and dried overnight. Fully reduced and permethylated glycans were used for downstream LC–MS analysis.

### 3.2. High-Resolution Mass Spectrometry (HRMS) Analysis of N-Glycans

LC–MS analyses were performed using an Ultimate 3000 nanoUHPLC system (Thermo Scientific, San Jose, CA, USA) coupled to an Orbitrap Fusion Lumos Tribrid mass spectrometer (Thermo Scientific, San Jose, CA, USA) equipped with a nano-electrospray ionization (nanoESI) source. Approximately 2 µg of permethylated N-glycans were injected and purified online using a C18 Acclaim PepMap 100 trapping column (75 µm × 2 cm, 3 µm, 100 Å; Thermo Scientific). Chromatographic separation was performed on a C18 Acclaim PepMap RSLC column (75 µm × 15 cm, 2 µm, 100 Å; Thermo Scientific) at 55 °C with a flow rate of 0.35 µL/min.

Chromatographic separation was performed using a gradient adapted from previously described LC–MS/MS methods for N-glycan analysis [24]. A 60 min chromatographic gradient was applied using mobile phase A (98% H_2_O, 2% acetonitrile, 0.1% formic acid) and mobile phase B (100% acetonitrile, 0.1% formic acid). The gradient program consisted of 20% B held for 0–10 min, followed by a linear increase to 42% B from 10 to 11 min, 42–55% B from 11 to 48 min, and an increase to 90% B at 49 min. The column was held at 90% B for 4 min and subsequently re-equilibrated at 20% B prior to the next injection.

Ion source settings. The instrument was operated in nano-spray ionization mode with static source gases. The spray voltage was +1600 V in positive ion mode and –600 V in negative ion mode. The ion transfer tube was maintained at 305 °C; no sweep gas was applied. The default charge state was 2, and source fragmentation was disabled.

MS acquisition. Full-scan MS spectra were acquired in the Orbitrap at a resolution of 120,000 (at *m*/*z* 200) over an *m*/*z* range of 400–2000. The AGC target was 4.8 × 10^5^ (normalized AGC 120%) with a maximum injection time of 50 ms. The RF lens was set to 30%. A minimum of 6 points per chromatographic peak was required. Only precursor ions with charge states 2–8 and a minimum intensity threshold of 19,000 were considered. Dynamic exclusion was applied after one occurrence for 20 s within a mass tolerance of ±10 ppm. Data were acquired in profile mode and positive ion polarity.

MS/MS acquisition. Data-dependent MS^2^ spectra (ddMS^n^) were collected in the ion trap at a rapid scan rate. Precursor isolation was performed with the quadrupole using a 2 *m*/*z* isolation window. Collision-induced dissociation (CID) was performed in assisted mode with stepped collision energies of 15%, 30%, and 45%, an activation time of 10 ms, and an activation q-value of 0.25. The AGC target was 1.0 × 10^4^ (normalized AGC 100%) with a maximum injection time of 35 ms. One microscan was acquired per spectrum. Data were recorded in profile mode and positive ion polarity.

### 3.3. Data Processing and Glycan Identification

Raw LC–MS data were processed using Xcalibur version 4.2 (Thermo Fisher Scientific) for chromatographic alignment, extraction of ion chromatograms, and verification of precursor masses and retention times [25]. Automated glycan identification and relative quantification were performed using the MultiGlycan software package version 1.4.0, which applies isotope deconvolution and charge-state recognition and compares identified ions with a curated N-glycan composition library. MultiGlycan integrates isotope envelopes across chromatographic peaks to calculate relative abundances and has demonstrated agreement within ±5 ppm when compared to manual validation [26].

## 4. Conclusions

This study demonstrates that sharp cheddar cheese contains Neu5Gc-containing N-glycans, whereas processed cheese slices show no measurable Neu5Gc species under the analytical conditions used. High-resolution nano-UHPLC–ESI–MS/MS successfully enabled the identification of multiple Neu5Gc-positive structures in cheddar cheese, including complex-type N-glycans such as HexNAc_4_Hex_4_Fuc_1_Neu5Gc_1_, which eluted between 25 and 36 min predominantly. The absence of Neu5Gc in processed cheddar cheese slices was observed under the applied analytical conditions; however, further studies are required to determine the relative contributions of formulation and processing parameters. Moreover, future research should be undertaken to study the mechanistic basis of Neu5Gc retention or loss during cheese production.

Although Neu5Ac-containing glycans were not assessed here, a broader comparative analysis of Neu5Gc versus Neu5Ac profiles across dairy products represents an important future direction for understanding the full spectrum of dietary sialic acids and their potential biological effects.

## Figures and Tables

**Table 1:** Identified N-glycans containing Neu5Gc in cheddar cheese.

Time (min)	Abundance (Area)	*m/z*	* HexNAc-Hex-deHex-NeuAc-NeuGc	Adduct	Monoisotopic Mass
31.03 ± 0.02	785,704 ± 311,042	1090.5680	3-4-1-0-1	[M+2H]^2+^;	2179.1197
31.75 ± 0.34	216,386 ± 86,570	1122.2480	7-5-1-0-1	[M+3H]^3+^;	3363.7222
32.43 ± 0.15	205,004 ± 78,406	887.1279	4-6-0-0-1	[M+3H]^3+^;	2658.3553
32.45 ± 0.01	282,013 ± 107,161	958.8302	5-5-1-0-1	[M+3H]^3+^;	2873.4706
32.73 ± 0.02	497,300 ± 143,293	949.4879	4-5-0-0-2	[M+3H]^3+^;	2845.4394
33.30 ± 0.03	3,317,978 ±1,277,497	1213.1300	4-4-1-0-1	[M+2H]^2+^;	2424.2455
35.44 ± 0.02	460,221 ± 183,735	1192.6180	3-5-1-0-1	[M+2H]^2+^;	2383.2190
36.24 ± 0.02	319,198 ± 83,108	1007.5170	4-5-1-0-2	[M+3H]^3+^;	3019.5282

* Glycan nomenclature: HexNAc—N-acetylhexosamines (GlcNAc or GalNAc), Hex—hexoses (mannose, galactose, or glucose), dHex—deoxyhexose (fucose—Fuc), NeuAc—N-acetylneuraminic acid, NeuGc—N-glycolylneuraminic acid.

**Table 2:** Cheddar cheese samples: CHE—sharp cheddar cheese (reduced fat) and SLI—sharp pasteurized prepared cheddar cheese product (slices). Both products were purchased from a local Walmart store in Lubbock, TX, USA.

Sample	Protein	Carbohydrate	Fat	Other Ingredients *
SLI—Sharp pasteurized prepared cheddar cheese product	14.3%	9.5%	21.4%	Water, cream, milk protein concentrate, whey, tricalcium phosphate, modified food starch, sodium citrate, citric acid, lactic acid, sorbic acid, color added, vitamin D3.
CHE—Sharp cheddar cheese (reduced fat)	28.6%	3.6%	21.4%	Color added, vitamin A palmitate.

* For both cheese products, the formulation includes a cheese base produced from pasteurized milk, starter cultures, salt, and enzymes. The “Other Ingredients” column lists additional components incorporated during processing, in accordance with product labeling.

## Data Availability

The original contributions presented in this study are included in the article. Further inquiries can be directed to the corresponding author.

## Abbreviations

The following abbreviations are used in this manuscript:

AGC	Automatic gain control
CID	Collision-induced dissociation
CHE	Sharp cheddar cheese
ddMS^n^	Data-dependent MS^2^ spectra
dHex	Deoxyhexose
Fuc	Fucose
GalNAc	N-Acetylgalactosamine
GlcNAc	N-Acetylglucosamine
Hex	Hexoses (mannose, galactose, or glucose)
HexNAc	N-acetylhexosamines (GlcNAc or GalNAc)
HPLC-ESI-MS/MS	High-performance liquid chromatography, electrospray ionization tandem mass spectrometry
HRMS	High-resolution mass spectrometry
ManNAc	N-Acetyl-D-mannosamine
*m*/*z*	Mass-to-charge ratio
nano-UHPLC–ESI–MS/MS	Nano ultra-high-performance liquid chromatography–electrospray ionization–tandem mass spectrometry
Nano-HPLC ESI-QTOF MS	Nano-HPLC coupled with quadrupole time-of-flight mass spectrometry (QToF MS)
Neu5Ac	N-acetylneuraminic acid
Neu5Gc	N-glycolylneuraminic acid
RT	Retention time [min]
SLI	Sharp pasteurized prepared cheese product
SNFG	Symbol nomenclature for glycans
LC MS/MS	Liquid chromatography–tandem mass spectrometry
q-value	Ion-activation q-value for ion-trap CID
QToF MS	Quadrupole time-of-flight mass spectrometry
WoS	Web of Science
